# Additively manufactured medical products – the FDA perspective

**DOI:** 10.1186/s41205-016-0005-9

**Published:** 2016-12-01

**Authors:** Matthew Di Prima, James Coburn, David Hwang, Jennifer Kelly, Akm Khairuzzaman, Laura Ricles

**Affiliations:** 1 US Food and Drug Administration, Center for Device and Radiological Health, Silver Spring, MD 20993 USA; 2 US Food and Drug Administration, Center for Drug Evaluation and Research, Silver Spring, MD 20993 USA; 3 US Food and Drug Administration, Center for Biologics Evaluation and Research, Silver Spring, MD 20993 USA; 4 US Food and Drug Administration, Office of the Commissioner, Silver Spring, MD 20993 USA

**Keywords:** Medical Device, Additive Manufacturing, Device Type, Final Device, Solid Oral Dosage Form

## Abstract

Additive manufacturing/3D printing of medical devices is becoming more commonplace, a 3D printed drug is now commercially available, and bioprinting is poised to transition from laboratory to market. Despite the variety of technologies enabling these products, the US Food and Drug Administration (FDA) is charged with protecting and promoting the public health by ensuring these products are safe and effective. To that end, we are presenting the FDA’s current perspective on additive manufacturing/3D printing of medical products ranging from those regulated by the Center for Devices and Radiological Health (CDRH), the Center for Drug Evaluation and Research (CDER), and the Center for Biologics Evaluation and Research (CBER). Each Center presents an overview of the additively manufactured products in their area and the specific concerns and thoughts on using this technology in those product spaces.

## Introduction

Recently, additively manufactured/3D printed medical devices have caught the world’s attention: a 3D-printed bronchial splint saved a child’s life [1, 2], a 3D printed cranial plate replaced a large portion of a patient’s skull [3], a new artificial knee was personalized to fit the patient’s own anatomy [4], and a spine device was made with complex internal architecture which was previously impractical to produce [5]. All of these are medical devices that have had profound effects on patient health and well-being. The FDA has been able to review and regulate these devices under existing regulations, by proactively identifying similarities with existing technologies and key differences that needed to be evaluated. For medical product production, additive manufacturing may offer an approach to make a device with complex architecture (e.g. integral porous coatings or internal lattice structure). Additionally, this technology has been leveraged to manufacture devices for specific patient’s anatomy (patient matching). While additive manufacturing of medical products has only recently entered the awareness of the mainstream media, the FDA’s Center for Device and Radiological Health (CDRH) has reviewed and cleared additively manufactured medical devices for more than 10 years. Over this time, there has been an increase in submissions utilizing this technology across a number of product areas; not just in medical devices but also including drugs and biologics. Recently, FDA’s Center for Drug Evaluation and Research (CDER) has approved a 3D printed drug and the Center for Biologics Evaluation and Research (CBER) has had interactions with stakeholders in the bioprinting field.

With the increase in utilization of additive manufacturing and the uncertainty of how the technology can affect the safety and effectiveness of the products, interest in additive manufacturing has significantly increased at the FDA. This interest led to the formation of the Additive Manufacturing Working Group to address these uncertainties and other questions about the additive manufacturing of medical products. The working group held a public workshop on October 8–9, 2014 to obtain input from stakeholders titled – Additive Manufacturing of Medical Devices: An Interactive Discussion on the Technical Considerations of 3D Printing [6]. The first day of the workshop aimed to bring together industry, academia, experts in the field and early adopting clinicians to discuss with the FDA the current state of the art for additive manufacturing, current best practices for validation and verification, as well as the technical challenges and associated solutions for additively manufacturing medical devices. The second day focused on the future use of bioprinting (3D printed tissue engineered biologics) and pharmacoprinting (3D printed pharmaceuticals). This editorial presents the current state of 3D printed medical products reviewed at the FDA, including drugs, biologics and devices. The primary focus is on the medical device industry, which have been early adopters. This editorial also provides a description of the regulatory pathways devices have taken and the regulatory considerations they face. Lastly, the outcomes of the technical workshop are presented, and how those technical considerations are being addressed since the workshop.

## Review

### Printing patient specific anatomy

The one aspect of 3D printing that has had a more immediate impact on the health care ecosystem has been the use of 3D printed anatomic models. There have been many publications regarding the use of printed anatomy in surgical planning, as a supplement to pictures from typical imaging modalities such as MRI, CT and X-Ray [7–10]. These uses have been particularly helpful with patients with unique anatomies, often in children who require complex surgeries due to anatomic anomalies [8, 9]. The 3D model of the patient specific anatomy has been reported by surgeons to better aid in visualization of the anatomy in question, when used with traditional images [11–13]. In this case, the 3D print is being used as a visual aid, and is treated much in the same way as a visible printed record of the anatomy (21 CFR 892.2040 Medical Image Hardcopy Device).

Software has been cleared through the 510(k) pathway that allows for segmentation of 3D patient scans, for example CT or MRI scans, to be converted to a 3D representation of the anatomy. This type of file, such as an STL, is similar to outputting an image as a PDF. The software used to generate the 3D model of the patient anatomy is evaluated by the FDA to assess the accuracy of the 3D volume reconstructed from image slices; however, the printer used to print the 3D component is outside of the scope of FDA review, much like an office’s laser printer would be when printing a PDF image. Prints of patient anatomy should be unaltered by the software if they are intended to be used for diagnostic or clinical purposes. If the patient’s anatomy is altered through the use of software in any way and indicated for diagnostic use, discussion with the Agency would be recommended. If patient specific anatomy is printed and then used specifically for designing a medical device (e.g. surgical cutting guide or implant), then the entire process, including the print, would be considered a device specific tool and should be part of the device submission (§201(h) Federal Food Drug & Cosmetic Act).

### Pharmacoprinting

FDA’s recent approval on a 3D-printed drug product in August 2015 (SPRITAM®) introduced a new chapter in pharmaceutical manufacturing for solid oral dosage form [14]. The principles of pharmacoprinting are essentially the same as other 3D printing types of medical products and can produce complex, personalized medicines on demand [15]. Personalized medicine began with the genomic revolution in the early 1990s [16], but current standardized dosage forms and associated manufacturing methods still have not achieved this objective. In addition, there are regulatory, legislative and process hurdles in fabricating a drug delivery system to meet the individual needs. Although the very first pharmacoprinted product approved by the FDA is a solid oral immediate release product, the majority of 3D printing research for oral delivery has been focused on controlled release, targeting and precise delivery for extremely low dose drugs. Like any other solid oral dosage forms, a 3D printed drug product must also be manufactured in accordance with current chemistry, manufacturing and control (CMC) standards as set forth in the 21 CFR 200 s & 300 s and other relevant guidance.

### Bioprinting

While there are currently no FDA approved or cleared biological products that incorporate additive manufacturing, the FDA, specifically CBER, has interacted with individuals who are using additive manufacturing technologies to print biological materials. Bioprinting offers many advantages over traditional tissue engineering techniques. For example, cells and biomaterials can be printed simultaneously with more precise spatial control in order to produce constructs with desired properties. Academic research published in the scientific literature demonstrates that bioprinting is an emerging field with a wide array of applications [17–20]. Specifically, additive manufacturing has been investigated for applications related to cellular and tissue constructs such as skin, cartilage, bone, nerve and blood vessels. However, applications related to bioprinting are largely still in the research & development phase.

### Medical device printing

Over the past decade CDRH has cleared dozens of additively manufactured devices through the 510(k) process [21] and in a few cases has approved them through emergency use [22]. While these devices were made using a variety of additive technologies and cover a number of device types, they can generally be separated into implantable and non-implantable devices and devices that are patient matched or non-patient matched. In addition to having the capacity to create patient-matched devices, additive manufacturing also provides the advantage of being able to create complex architectures. For example, some orthopedic implant manufacturers leverage this technology to create a complex porous structure integrated with the solid parts of the device (e.g. acetabular shell with integral porous coating) and others are composed of porous or lattice structures (e.g. bone wedges or spinal cages). Implantable devices such as joint replacements, cranial implants, maxillofacial implants and restorative devices such as dental crowns and bridges may also be matched to the patient’s anatomy for a variety of clinical needs. Additive manufacturing is also commonly used to manufacture patient matched cutting guides/drill templates, which are non-implantable products that are used during surgery (e.g. for knee, ankle, shoulder and maxillofacial surgeries).

Most of the additively manufactured devices just described have been reviewed as Class II devices through FDA CDRH’s Premarket Notification (510 (k)) Program. The classification regulations are based on the risks to health for a device’s intended use and the level of control necessary to provide for a reasonable assurance of the safety and effectiveness of the devices. Depending on the device type, the use of additive manufacturing may present additional technical challenges in terms of manufacturing controls, device performance, biocompatibility and sterilization, etc. It does not, in general, raise new questions of safety or effectiveness. Therefore, unless an additively manufactured device presents a new or different question of safety or effectiveness for its device type, the device would be classified into the same regulatory class as other devices of that type, regardless of manufacturing method.

There are a couple of points that the FDA would like to clarify that are commonly misunderstood by medical product device stakeholders, especially in regard to additive manufacturing. First, there is often a misunderstanding that FDA clears or approves materials for various medical uses. Rather, CDRH evaluates a material within the context of the technological characteristics of the device along with the intended use and determines if the device’s intended use and technological characteristics (including the materials) are substantially equivalent in safety and effectiveness to a legally marketed device. If so, FDA provides clearance to individual devices for specific intended uses, not to materials for unspecified intended uses. Devices containing new materials may be cleared through the 510(k) process provided that the new material does not raise new questions of safety or effectiveness and the submission demonstrates that the new material is at least as safe and effective as those in an equivalent legally marketed device. However, it may be helpful in a submission to identify a predicate or previously approved device that incorporates the same material as your device, as this comparison may help in your effort to demonstrate substantial equivalence. The concept is the same for additively manufactured devices. FDA can clear a specific device made by additive manufacturing for a specific intended use but it does not separately clear or approve raw material stock, final printed material or a printing process (e.g. Ti6Al4V using electron beam melting) for unspecified uses. However, predicate devices using the same additive manufacturing process may reduce a submission’s premarket burden by leveraging previously provided information or testing.

Second, another source of confusion that the FDA would like to clarify is the concept of “custom” versus “patient matched” devices. Colloquially, “custom device” has been referred to as a device that is made specifically for one application or in this context, a specific patient; however, the use of “custom” has a specific regulatory definition in Section 520(b) of the Federal Food, Drug and Cosmetic Act (FFD&C Act), which is further discussed in FDA’s Guidance titled “Custom Device Exemption – Guidance for Industry and Food and Drug Administration Staff.” The guidance provides definitions of terms used in the custom device exemption, explains how FDA interprets the “5 units per year of a particular device type” language contained in section 520(b)(2)(B) of the FD&C Act, describes what information should be submitted in a Custom Device Annual Report (“annual report”), and provides recommendations on how to submit an annual report for devices distributed under the custom device exemption. The guidance states as follows:

*“It is worth noting that FDA reviews, clears and approves for marketing many patient-specific devices (also referred to as patient-matched devices). Patient -specific devices are, in general, ones in which ranges of different specifications have been approved or cleared to treat patient populations that can be studied clinically. Premarket submissions for such devices are sometimes referred to as “envelope” submissions because their approval or clearance covers the entire range of specifications data they contain to support. The final manufacturing of these devices can be delayed until physicians provide imaging data or other information to the manufacturer to finalize device specifications within cleared or approved ranges. As a result, such devices are specifically tailored to patients. For example, a manufacturer of an ankle replacement device could submit a 510(k) to cover a range of specifications for different system components to accommodate multiple patients with different anatomical characteristics. While some in industry have sometimes colloquially referred to these devices as “customized,” they are not custom devices meeting the FD&C Act custom device exemption requirements unless they comply with all of the criteria of section 520(b). Marketing applications are required for these device types because the devices and patient populations can be defined and studied.”* [23].

Despite some new technological characteristics of additive manufacturing, this has not changed the regulatory pathway for medical products that are reviewed by the FDA. This not only applies to premarket review, but also to manufacturing quality during production.

Unless specifically exempted, all devices, including additively manufactured ones, must comply with the same manufacturing quality and compliance requirements under 21 CFR 820, also known as the Quality System regulations (QSr), where current good manufacturing practice (cGMP) requirements are set forth. The regulation applies to so many different types of devices that it cannot prescribe specific details for each process. Instead, it provides a framework for manufacturers to establish and follow quality systems that will help ensure their products consistently meet applicable requirements and specifications. The requirements in this part are intended to ensure that finished devices will be safe and effective and otherwise in compliance with the FD&C Act. Established medical device manufacturers will be familiar with typical methods used to meet the QSr, but some of the practices may be new to start-up companies or recent entrants to the medical device field.

Newcomers to the medical device manufacturing area may find CDRH’s Division of Industry and Consumer Education (DICE)^1^ a valuable resource. DICE was created specifically to provide technical and regulatory assistance to small manufacturers to help them comply with the regulatory requirements for medical devices. The Division also assists large manufacturers, academia and research organizations, consultants, attorneys, customs brokers, government agencies, user facilities, individuals who invent or market devices, among others. For questions relating to biological products, CBER’s Manufacturer’s Assistance and Technical Training Branch (MATTBB)^2^ is also a useful resource. MATTBB provides training and assistance to industry and responds to requests for information regarding CBER policies and procedures. For questions related to drug products, CDER’s Division of Drug Information (DDI) 3 is available to provide expert advice and guidance regarding all aspects of the Center’s activities.

### FDA’s 2014 workshop

FDA has shown through experience that additive manufacturing follows the same regulatory pathway and manufacturing requirements as non-additively manufactured devices. However, just as each manufacturing method (e.g. casting, injection molding, machining) have their own specific technical aspects and considerations, so does additive manufacturing. The 2014 Public Workshop brought together a broad spectrum of stakeholders to discuss these technical considerations. Discussions were separated into 5 broad technical themes: (1) materials, (2) validation for design, printing and post printing, (3) printing characteristics and parameters, (4) physical and mechanical assessment of final devices, and (5) biological considerations of final devices, including cleaning, sterility and biocompatibility. To facilitate and provide perspective, a panel of external experts held a moderated discussion with the attendees on each theme, starting with several seed questions from the FDA. Even though each theme was discussed separately, there was a common understanding that the final device quality and performance strongly depended on the interplay between all the themes.

Several of the most critical factors to device quality rose to the top: 1) Build orientation and location can affect final device performance. Geometric features will often have different mechanical and material properties based on their location in the build volume and orientation of print. This can be especially important with small features such as porosity or load bearing features. 2) Validation of additive manufacturing systems in the location they will be used is key to achieving success. Moreover, workshop experts agreed that it takes one to two years of experience using a specific process and printer combination to build confidence in the procedures. There are many ways to perform quality assurance as well. Some companies stated that they only produced one model of a product per machine. Others used process validation principles and advanced monitoring or verification techniques to produce multiple models in one machine. Whichever quality assurance protocols were chosen, it was clear that every machine is different and needs to be validated and monitored individually. 3) Patient matching is one of the greatest design advances enabled by additive manufacturing. It brings personalized medicine to medical devices. Unlike traditional designs that can go through a final design review once, these patient matched devices are all slightly different. Instead of reviewing one final design, the entire design envelope (i.e. the range of each parameter that can be modified), patient-matching processes and identification procedure must be completed. 4) Sterilization of additively manufactured devices is fundamentally no different than for other devices. Small design features or internal structures may make location of test samples more difficult. Cleaning on the other hand, can be a challenge for additively manufactured devices with complex or small structures. Powder bed and liquid bath based systems all have residual raw material that must be removed before the device can be used. Measuring when it has been removed can be more difficult for parts with small intricate features. After the initial cleaning, if any machining or other manufacturing steps are taken, those residues must also be removed from the same small, intricate spaces. Many attendees, including those from other industries agreed that these considerations are extremely important when building an additively manufactured product but that they do not create insurmountable problems.

While the workshop served as an important starting point in the discussion on the technical considerations of additive manufacturing of medical devices, the FDA’s efforts have not ended with the workshop. Since last October, the FDA has participated in a number of conferences and forums discussing additive manufacturing [24, 25] and has continued the conversation with specific stakeholders during the review of their devices. From these interactions, along with research efforts within CDRH, we have expanded our knowledge base and research experience [26, 27]. FDA and device manufacturers continue to work together to ensure new, innovative and personalized devices can be safely and effectively made through additive manufacturing.

## Conclusion

The FDA has continued its mission of ensuring patients and providers have access to safe and effective medical products while endeavoring to provide industry with a predictable, transparent and efficient regulatory pathway for additively manufactured devices. The Center for Drug Evaluation and Research approved the first 3D printed drug within the existing chemistry, manufacturing and control standards that all other drug products are regulated by. The Center for Devices and Radiological Health have cleared additively manufactured devices for over a decade within the existing medical device regulations. The Center for Biologics Evaluation and Research and the other Centers are following the literature closely and are interacting with stakeholders to ensure that US patients have access to innovative, safe and effective medical products as this technology expands.

## Footnotes

1 Email: DICE@fda.hhs.gov; Phone: 1(800) 638–2041 or (301) 796-7100

2 Email: industry.biologics@fda.gov: Phone: 1 (800) 835-4709 or (240) 402-8010

3 Email: druginfo@fda.hhs.gov: Phone: 1 (855) 543-3784 or (301) 796-3400
